# Photon shifting and trapping in perovskite solar cells for improved efficiency and stability

**DOI:** 10.1038/s41377-024-01559-2

**Published:** 2024-09-05

**Authors:** Sirazul Haque, Miguel Alexandre, António T. Vicente, Kezheng Li, Christian S. Schuster, Sui Yang, Hugo Águas, Rodrigo Martins, Rute A. S. Ferreira, Manuel J. Mendes

**Affiliations:** 1 CENIMAT|i3N, Department of Materials Science, School of Science and Technology, NOVA University of Lisbon and CEMOP/UNINOVA, Campus de Caparica, Caparica, Portugal; 2 Department of Physics and CICECO - Aveiro Institute of Materials, University of Aveiro, Campus Universitário de Santiago, Aveiro, Portugal; 3 Materials Science and Engineering, School for Engineering of Matter Transport and Energy, Arizona State University, Tempe, AZ USA; 4 Department of Physics, University of York, Heslington, York UK

**Keywords:** Solar energy and photovoltaic technology, Optoelectronic devices and components

## Abstract

Advanced light management techniques can enhance the sunlight absorption of perovskite solar cells (PSCs). When located at the front, they may act as a UV barrier, which is paramount for protecting the perovskite layer against UV-enabled degradation. Although it was recently shown that photonic structures such as Escher-like patterns could approach the theoretical Lambertian-limit of light trapping, it remains challenging to also implement UV protection properties for these diffractive structures while maintaining broadband absorption gains. Here, we propose a checkerboard (CB) tile pattern with designated UV photon conversion capability. Through a combined optical and electrical modeling approach, this photonic structure can increase photocurrent and power conversion efficiency in ultrathin PSCs by 25.9% and 28.2%, respectively. We further introduce a luminescent down-shifting encapsulant that converts the UV irradiation into Visible photons matching the solar cell absorption spectrum. To this end, experimentally obtained absorption and emission profiles of state-of-the-art down-shifting materials (i.e., lanthanide-based organic-inorganic hybrids) are used to predict potential gains from harnessing the UV energy. We demonstrate that at least 94% of the impinging UV radiation can be effectively converted into the Visible spectral range. Photonic protection from high-energy photons contributes to the market deployment of perovskite solar cell technology, and may become crucial for Space applications under AM0 illumination. By combining light trapping with luminescent downshifting layers, this work unravels a potential photonic solution to overcome UV degradation in PSCs while circumventing optical losses in ultrathin cells, thus improving both performance and stability.

## Abstract (web summary)

Combining a simple (yet powerful) light-trapping structure with a luminescent down-shifting material (t-U (500)/Eu3 + ) allows remarkable efficiency enhancement (28%) in perovskite solar cells while providing protection against damaging UV radiation.

## Graphical abstract

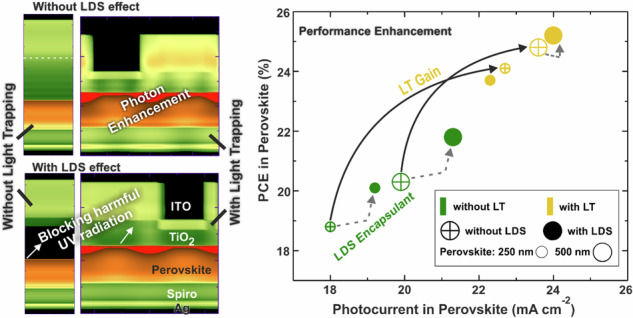

## Introduction

Perovskite-based materials have been a central focus of research in highly efficient photovoltaic (PV) technologies. Their exceptional optoelectronic properties enabled perovskite-based solar cells to achieve remarkable growth in power conversion efficiency (PCE) in 12 years, going from 3.8% to 26.1%^1,2^, which is comparable to the efficiency of contemporary commercial c-Si solar cells. Most importantly, perovskite solar cells (PSCs) have the potential to become the low-cost market leader in consumer-oriented solar-powered systems (EVs, BIPV, portable electronics, IoT, etc.)^3^ if their lifetime can be increased from around one year to 15+ years. Nevertheless, the lifetime of PSCs is affected by extrinsic (environmental) and intrinsic factors. Environmental factors such as moisture and oxygen can be solved by encapsulation, whereas intrinsic factors, such as hygroscopicity, thermal stress, and ion migration, lead to instabilities within the perovskite bulk material and its interface to the charge transport layers^4^.

UV degradation, for instance, is one of the most detrimental intrinsic factors as it leads to irreversible degradation of perovskites upon formation of I_2_ or polyiodide I_3_^-^ (ref. ^5^). Such issue becomes particularly critical in the realm of new Space applications, where the paramount irradiation test for devices revolves around their resilience to UV exposure. This is especially crucial due to the exceptionally high levels of UV radiation encountered under AM0 conditions^6^. The degradation is typically triggered by interfacial photocatalytic reactions between electron transport layer (ETL) in high-performing PSCs and the perovskite film^7,8^. This photostability problem can be easily solved if UV radiation does not reach the interface of ETL/perovskite layers. However, by using a UV-shielding encapsulant layer in PSCs^8,9^, the UV photon of the solar spectrum will be lost, hence limiting further efficiency gains. Recently, there has thus been an increasing trend towards engineering a layer that not only functions as an encapsulant, but also as a photon-recycler for the incident UV light.

By adding luminescent down-shifting (LDS) fluorophores to the encapsulant layer^8,10–13^, one can protect the PSCs from environmental factors and convert high energy photons into lower energy ones. These lower energy photons can then be absorbed by the perovskite layer without issues, and the increased number of visible photons available for absorption by the cell will in turn increase the external quantum efficiency (EQE) of the PSC^8^. Among distinct luminescent materials^14^, such as organic dyes^15–17^, quantum dots (QDs)^15,16,18^, and lanthanide metal ions/complexes^18–22^, the lanthanide-based beta-diketonate complexes stand out. The well-known Ln^3+^ luminescence sensitization, or antenna effect, can be used as an efficient light-conversion molecule. The ligand-induced large Stokes’ shift and the ligand-to-Ln^3+^ energy transfer processes ensure an efficient UV-downshifted emission towards the visible spectral range^23^. Lanthanide (Ln^3+^) based materials, such as Eu or Tb, have shown significant potential for acting as UV absorbers in the 300 to 400 nm wavelength region, and for efficiently shifting these impinging photons into useful lower-energy photons in the visible region^13,24–28^. In addition, the LDS layers composed of such Eu^3+^ and Tb^3+^ materials manifest stronger ligand-induced Stokes shifts compared to other LDS materials, such as organic dyes or QDs, thus posing smaller losses from self-absorption^28^. LDS layers made of doped organic-inorganic hybrid materials with Eu^3+^ and Tb^3+^ have already been implemented on PV devices; for instance, c-Si-based PV cells displayed an absolute EQE increase of ∼27%^28^. Recently LDS materials have also been applied in PSC technology to improve the PV response while protecting the cells from UV-induced degradation^29–31^. In particular, Rahman et al^31^. demonstrated *a* ~14% enhancement of PCE in PSCs in combination with improved device stability by using LDS layers composed of Eu^3+^.

Enhancing the material quality of perovskite PV to improve carrier mobility and minimize defect density is another crucial aspect for achieving high performance. One way to reduce bulk recombination is to reduce the thickness of the active layer. Yet, thin absorbers suffer from reduced light absorption, hence limiting their efficiency^32^. Advanced light-management techniques, particularly light-trapping (LT) solutions, can offer a promising solution to increase the optical path within the active layer and thereby absorb more light ^33^.

Light trapping serves as a key strategy to attain physically thin but optically thick absorbers that not only results in mutual benefits for both optical and electrical properties^34–36^, but also reduces material consumption (especially hazardous/toxic elements, such as Pb, present in common perovskite compositions)^37^ and fabrication costs. This LT strategy may even lead to the increased mechanical bendability of the devices ^38^.

Recent studies have highlighted the particularly promising potential of simple grating structures for LT in PSCs^39^. They have also been shown to achieve photocurrent enhancements in thin-film c-Si comparable to more complex photonic strategies like quasi-random super-cell structures^40,41^. These structures may result from the displacement and rotation of periodic grating lines, yielding a trellis pattern that resembles a checkerboard. One notable advantage of periodic grating lines is their industrial-friendly integration, which is conducive to large-scale applications^40^. This makes them highly appealing for practical implementation in the manufacturing of PSCs, as they offer a feasible and scalable approach to enhance light absorption and boost the overall performance of the cells. As such, using a simple trellised structure represents a promising avenue for advancing the PSCs design and unlocking their full potential for efficient and cost-effective solar energy harvesting.

By endowing PSCs with robust encapsulant properties and advanced light-management architectures, new areas of application open up for this PV technology. Among the most relevant areas are Space applications, where PSCs can be a viable alternative to the III-V multi-junction solar cells (CdTe, GaAs, CIGS) commonly used in this field, owing to their potential for high efficiency at a lower weight and cost. The lightweight and flexible nature of PSCs can reduce the overall weight of solar panels, which is critical for Space missions. Furthermore, the potential for high power-to-weight ratio^42^ and flexibility of PSCs offers new opportunities for innovative design and integration in Space systems. For instance, their thin-film nature and solution processability allows for conformal coating on various substrates, such as flexible and curved surfaces, which can enable novel solar panel configurations and maximize the use of sunlight-exposed surfaces on satellites and spacecraft. Moreover, some of the environmental stressors that reduce the lifetime of the PSCs are attenuated in Space where oxygen and moisture barely exist. What’s more, PSCs have shown remarkable radiation tolerance and self-healing capabilities, with studies demonstrating that they can maintain their high efficiency even after exposure to high doses of radiation ^43,44^.

This work now demonstrates a novel strategy for significantly boosting the sunlight-to-electricity conversion of PSCs while greatly improving their UV stability and flexibility. To achieve that, coupled optical and electrical modeling, grounded on experimental results, was employed to explore a combination of two unprecedented optical approaches in PSCs: constructing a distinctive photonic checkerboard design for LT combined with photon down-shifting. First, the checkerboard (CB) gratings were arranged with symmetry-property and optimized for integration as an LT structure for PSCs. Secondly, we studied the coupling of the optimized CB photonic front structure with an LDS encapsulant material composed of an experimentally developed tri-ureasil modified by lanthanides (t-U (5000)/Eu^3+^). Both approaches lead to substantial performance improvements in PSCs. Aside from significant photocurrent enhancement, the LT-enhanced ultrathin PSCs showed improved open-circuit voltage and fill factor, resulting in a significant PCE enhancement of 28%. Subsequently, it was found that the investigated LDS layer blocks nearly 94% of the total UV radiation in the 300–400 nm spectral region, re-emitting it into the visible spectrum, which translates to electrical gains for the PSC. This combined optical strategy in PSCs not only opens up a wide range of PSC applications in consumer electronics devices, but it also bodes well for the next generation of high-efficiency Space solar technology.

## Results

**Fig. 1:**
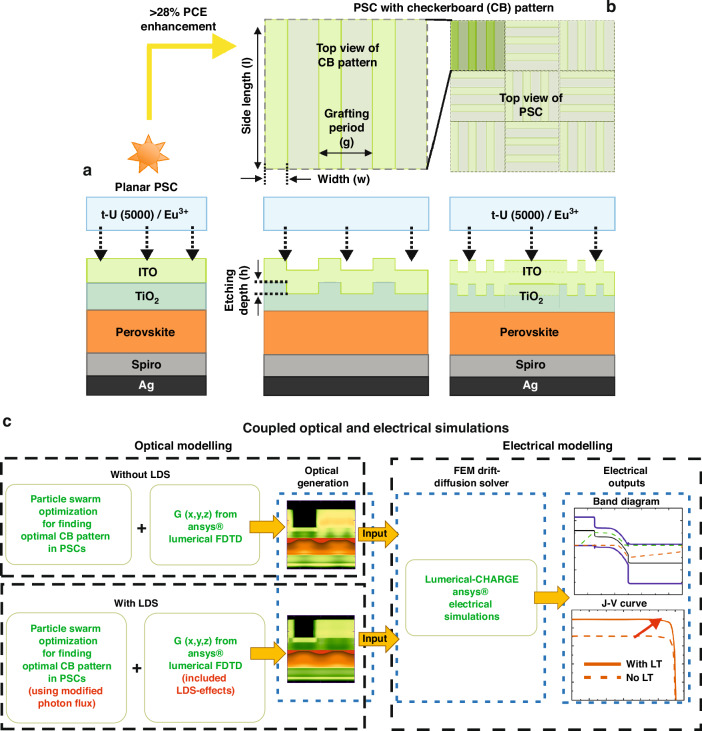
Solar cells design endowed with photon trapping and/or down shifting. Sketch of the architecture of planar (**a**) and photonic-enhanced (**b**) PSCs with an LDS encapsulant coating composed of t-U (5000)/Eu^3+^. The innovative LT design applied on the front contact of the PSCs (**b**) consists of periodic grating lines that form a trellised checkerboard (CB) pattern in the TiO_2_ electron transport layer (ETL). The geometrical parameters (*h, w, g, l*) for the CB patterns (**b**) considered for optimization are indicated with arrows. Two distinct thicknesses (250 nm and 500 nm) were selected for the perovskite absorber layer. The thicknesses of the remaining layers in the PSCs are indicated in Table S1 in Supplementary Material (SM). **c** depicts the flow of the coupled optoelectronic simulations used in this study. To incorporate the LDS effect, the Lumerical built-in FDTD global AM1.5/AM0 spectrum was modified to incorporate the absorption and emission flux of the experimentally developed t-U (5000)/Eu^3+^ before modeling the PSCs with the LDS encapsulant layer

A rigorous coupled optical and electrical model was developed to simulate and optimize the response of PSCs endowed with LT and LDS effects, which is sketched in Fig. 1 and described in section “Materials and methods”.

This section starts with a comprehensive optical analysis of the optimized CB pattern for PSCs (section “Optical performance of LT-enhanced PSCs”), followed by the electrical analysis in section “Electrical performance of LT-enhanced PSCs”, namely band-diagrams, J-V curves, and main PV quantities as short-circuit current (Jsc), open-circuit voltage (Voc), Fill-Factor (FF) and power conversion efficiency (PCE). The CB grating structures are top-coated as a final processing step, which prevents the structuring of the active absorber layer, which could lead to electrical degradation via increased recombination^45^. On a side note, the proposed CB design can be fabricated in a highly scalable and high-throughput fashion, for instance by nano-imprint^39,46,47^ — a high-resolution and low-cost soft micro-patterning method compatible with PSCs. Laser Interference Lithography (LIL) is another promising fabrication process for the CB structures^48^. However, the CB structure would require a contact mask (to define cut lines) and two exposures (to define horizontal and vertical lines), which involves a 90° sample rotation. In contrast, Displacement Talbot Lithography (DTL)^40^ offers notable advantages. Firstly, features are scaled up by a factor of two on the mask, simplifying the fabrication process. Secondly, all horizontal and vertical lines are simultaneously exposed, streamlining the production. Thirdly, the proximity of the mask to the sample in DTL ensures a secure mask use for multiple exposures, along with enhanced stability of the interfering beams compared to LIL.

Section “Luminescent down-shifting properties in PSCs” focuses on the optical performance of the t-U(5000)/Eu^3+^ LDS, with and without CB patterns (section “Optical performance of LDS in PSCs”), and, lastly, on the overall performance of CB and t-U(5000)/Eu^3+^ LDS in PSCs (section “Performance analysis of LT and LDS in PSCs”).

### Photonic-enhanced PSCs with checkerboard pattern

#### Optical performance of LT-enhanced PSCs

**Fig. 2:**
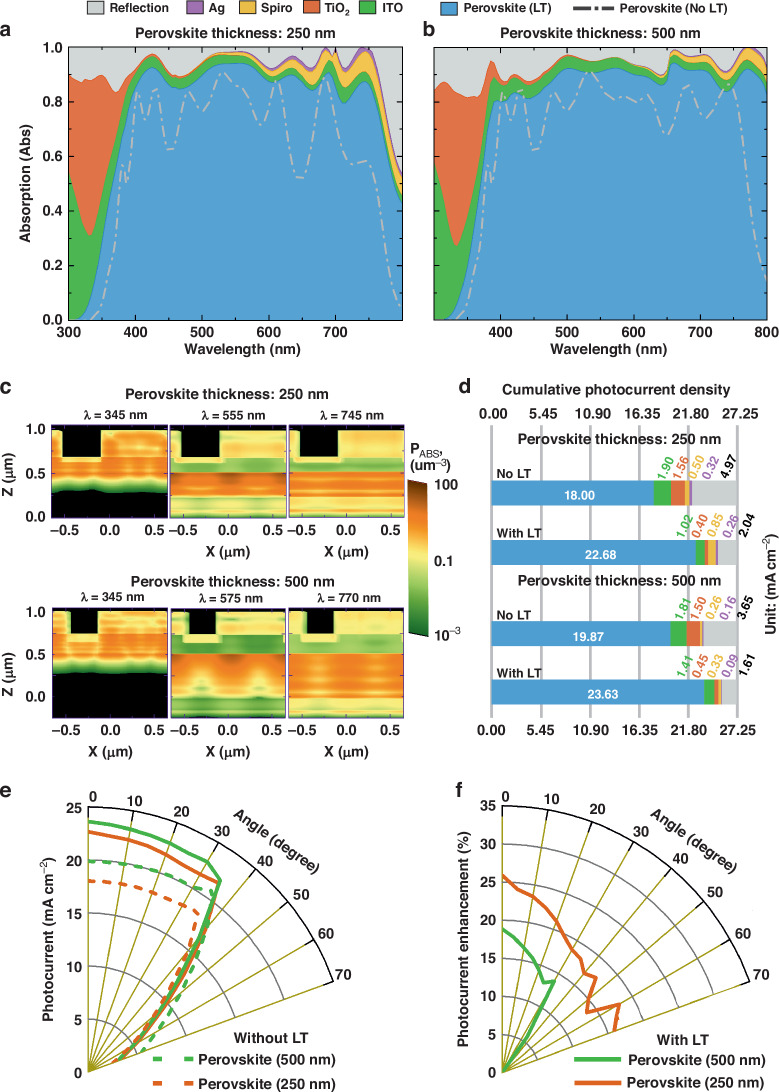
Optical response of LT-structured PSCs, relative to planar devices. **a**, **b** Absorption spectra attained with the optimized CB pattern in PSCs (without LDS layer), and with the reference cases of flat cells with planar ITO and TiO_2_ layers, for perovskite absorber layers with distinct thickness: (**a**) 250 nm and (**b**) 500 nm. Colored regions show the absorption in different materials as well as the reflection losses for the optimized CB pattern, and the gray lines depict the absorption that occurred only in perovskite for the reference cases of planar cells (No LT). **c** Log-scale distributions of the light absorption density, *p*_*ABS*_, along the *xz* plane of the CB arrangement passing through the center of the *y* plane, for selected wavelengths (λ), for both 250 nm and 500 nm perovskites. **d** The cumulative photocurrent density was calculated from the absorption spectra, for the cells with and without the LT structures. The different colors correspond to the optically-inferred photocurrent at the materials indicated in **a**, **b**. **e** Angle-resolved unpolarized photocurrent density, *J*_PH_, determined for both planar and LT cells with 250 nm and 500 nm perovskite thickness, and (**f**) shows the photocurrent improvement achieved with LT with respect to the planar reference in the same angular range

Figure 2 summarizes the results of the optical analysis of the photonic-enhanced PSCs, considering the geometrical parameters described in Fig. 1b. The thicknesses of the PSC layers for the thin conductive oxide (TCO), the electron- (ETL) as well as hole transport layer (HTL), and metal contact (respectively t_ITO_, t_TiO2_, t_Spiro_, and t_Ag_) were restricted to feasible fabrication settings indicated in Table S1 in SM. For both an ultra-thin (250 nm) and a conventional perovskite active layer (500 nm), the results of the photonic-enhanced PSC are compared to a reference device, consisting of planar ETL (TiO_2_) and TCO (ITO) layers with the same thicknesses as the CB-patterned PSC (see Fig. 1a). We choose TiO_2_ as the ETL for its widespread use in state-of-the-art PSCs and its optical advantages as a LT medium, attributed to its favorable refractive index properties^49^. Further details regarding the choices of layer thicknesses for the selective contacts are provided in section S1 in SM.

Figure 2a, b depict the absorption profiles for the reference planar cells as well as LT-enhanced cells, with 250 nm and 500 nm thickness perovskite active layer. Here, two spectral regions of interest can be emphasized. Firstly, in the UV range (300–400 nm) all cells show low absorption in the perovskite, mostly due to reflection losses and parasitic absorption from the front contact. Secondly, in the visible range (400–700 nm) the devices show peak absorption, and the contribution of the LT structures to enhancing the photocurrent density (*J*_PH_) is highest here, with an absorption of consistently over 80%. For the perovskite layer thickness of 250 nm and 500 nm, the *J*_PH_ gain due to the LT structures is 26% and 19%, respectively, see Fig. 2d. Therefore, the thinner the active layer, the more advantageous the addition of LT structures can be. This is particularly true for the poorly absorbed wavelengths above 600 nm, as these require longer optical paths to contribute to photocurrent generation. The observed *J*_PH_ gain is mainly attributed to the anti-reflective (AR) effect and strong light scattering due to the broken symmetry of the quasi-random photonic patterning of the optimized CB arrangement:

- The better index matching suppresses unwanted reflections,
- There is a strong in-coupling of light towards the perovskite,
- The optical path lengths become longer as the light is diffracted obliquely into the absorber.

Together, these mechanisms can strongly improve broadband absorption. This is visualized by the power absorption density profiles shown for different wavelengths in Fig. 2c. The cumulative photocurrent densities also show a significant decrease in overall reflection for the 250 nm cell (from ~5 to ~2 mA cm^−2^) in comparison to the 500 nm cell (from ~3.7 to ~1.6 mA cm^−2^), see Fig. 2d.

Essentially the broken symmetry is explained via the Fast Fourier transform (FFT) of the structures as explained in section S2 of SM. For instance, periodic gratings excite a well-defined Fourier spectrum, where the peaks correspond to the main frequencies of the structure. Optically, this corresponds to high-intensity scattering into specific directions. Contrarily, random surface roughness excites numerous diffraction orders due to their broken symmetry, thus it creates lower intensity scattering for a broader range of directions. As such, the optimal light-trapping structure lies between these two extremes, allowing for both broadband absorption and enough degrees of freedom to make the structure experimentally achievable^40^. Considering this, we enhanced the randomness by breaking the symmetry while preserving the periodicity by expanding the size of the unit cell in our CB design. The high number of finger lines in the checkerboard design allows better index matching, resulting in strong anti-reflection (light in-coupling) properties (consequently, high absorption in shorter wavelengths of 300 to ~500 nm as seen in Fig. 2a, b), while a larger grating period provides strong light scattering (consequently, high absorption in longer wavelengths of ~500 to 800 nm as also seen in Fig. 2a, b).

Since the photonic structures in the solar cells are optimized for maximum broadband absorption rather than resonance at a specific wavelength, the resulting absorption curves with LT could be comparable for the two perovskite thicknesses. Nonetheless, the degree of absorption above 650 nm wavelengths differs significantly between the 250 nm and 500 nm perovskites, as seen in Fig. 2a, b, even more so for the planar cases (see dash-dot spectra) without LT. The absorption curves for 500 nm perovskite are much broader in these wavelengths, whereas for 250 nm the NIR peaks are relatively narrow and drop significantly above 750 nm.

Table S1 displays the optimal geometrical characteristics of the CB patterns (*h*, *w*, *g*, *l*) that maximize the photo-generation in the perovskite layer for the different cases under study. Analysis of this table allows us to make the following statements.

When no LDS coating is applied (background index *n* = 1), the thinner PSCs (250 nm absorber) require greater etching depth (*h*) and narrower fingers (*w*), whereas thicker PSCs (500 nm) prefer shorter and wider geometries. This trend is similar to that observed when optimizing front photonic features for thin silicon solar cells^35,50^. The thicker perovskite benefits from a more delocalized spread of the scattered light; therefore, higher scattering cross sections of the front features are preferred, which are achieved by structures with a *lower* aspect ratio. The thinner perovskite instead benefits from a more localized (focused) spread of the scattered light, achieved by structures with a *higher* aspect ratio. The FFT analysis (Fig. S3 in SM) indicates the different scattering modes to which light can be dispersed. The FFT results also support the previous reasoning behind the observed *J*_PH_ gain from the LT structures, especially at longer wavelengths, where the limited horizontal maneuvering of light in the thinner device hinders the effective scattering of light. From Fig. S3, one can see that the thinner perovskite has much lower lateral light scattering compared to the 500 nm cell.

When the LDS coating is applied (background index *n* = 1.5), as analyzed further below in section “Luminescent down-shifting properties in PSCs”, the larger *n* value reduces the index contrast with the CB structure and thus considerably weakens the scattering cross-sections of the front features. As such, it becomes preferable that the 500 nm perovskite attains its LT gain via a better in-coupling (anti-reflection) of the LDS-enhanced visible light, which also justifies the shift towards narrower CB features with higher aspect ratio.

For the optimized planar and photonic-enhanced devices, Fig. 2e, f shows how the photocurrent varies with the angle of incidence. The most relevant observation from Fig. 2e is the almost constant photocurrent response, which only experiences a significant drop at an angle ~40°. This is mainly due to Brewster-like reflection effects on the flat front surfaces which cause a sharp increase in reflection at higher incident angles, as explained elsewhere^43^. Besides, since the in-coupling and light scattering provided by the CB geometry was not optimized for oblique incidence, a pronounced angular decrease of the *J*_PH_ gains (Fig. 2f) is not unexpected. Nevertheless, the higher aspect ratio structures on the thinner 250 nm perovskite can better overcome such reflection losses at high angles, thus avoiding a large drop in the LT enhancement as observed with the thicker 500 nm perovskite.

Lastly, we point out that for all angles of incidence the ultrathin 250 nm cell with LT outperforms the conventional 500 nm cell (planar, without LT) in photocurrent output, which demonstrates that an effective photonic scheme is a promising means to compensate for smaller absorber thicknesses. We also note that reducing the perovskite thickness by half can potentially offer a 3-fold improvement in device flexibility, a significant reduction in material costs, and a 2-fold reduction in toxic lead (Pb) use.

#### Electrical performance of LT-enhanced PSCs

**Fig. 3:**
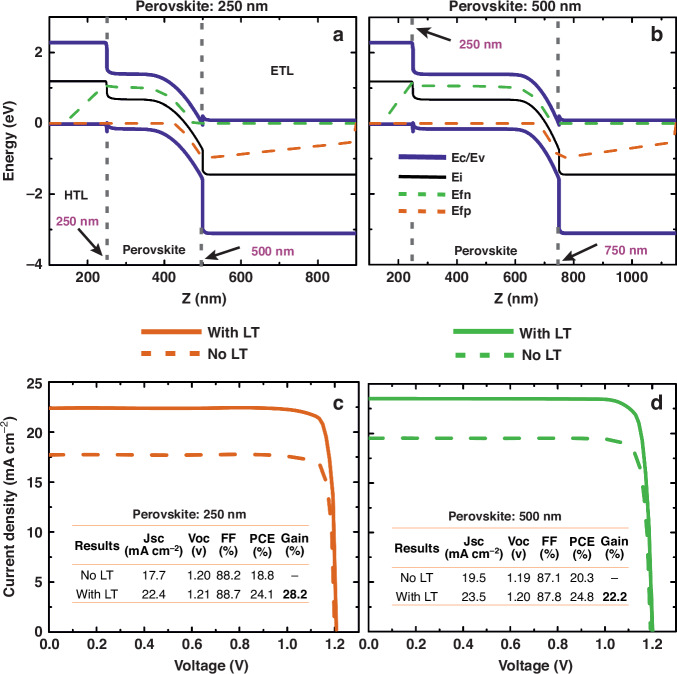
Electrical results with drift-diffusion model. **a**, **b** show the band diagram for the PSC with 250 nm and 500 nm perovskite thickness, where the location of the perovskite layer is indicated by the dashed lines. **c**, **d** display the J(V) characteristics of the planar and photonic-enhanced PSCs. The inset tables present the main PV quantities extracted from the curves

The electrical results of the optimized structures described above are summarized in Fig. 3. Figure 3a, b shows the overall band structure for both perovskite thicknesses, from which the main transport properties of the devices can be inferred. For instance, in the 500 nm perovskite case, the constant E_C_/E_V_ region within the perovskite layer (*Z* ~ 300–600 nm) reveals a weak electric field (almost constant potential), that could negatively affect the charge transport since the drift component is small^51^. Yet, this effect turns out to be negligible. It only has a small impact on the fill factor of the 500 nm cell compared to the 250 nm cell (87.8% and 88.7%, respectively), because the perovskite’s layer thickness is still much smaller than the diffusion lengths of the charge carriers^52^. It is important to note that the electric field remains largely unchanged when adding the LT structures, as it directly depends on the intrinsic properties of the material used.

According to Fig. 3c, d, the *J*_SC_ values decrease compared to their optical counterparts, *J*_PH_ (presented in Fig. 2), after including recombination losses into the electrical model. Nonetheless, the LT-enhanced devices show a significant increase in *J*_SC_. Given its minor influence on the V_OC_, this boost can be considered the main cause of the PCE enhancement relative to the planar case (28% for the 250 nm cell and 22% for the 500 nm cell). The LT application results in modest electrical gains, with approximately 0.8% and 0.5% increases in Voc and FF, respectively, observed for the 250 nm and 500 nm perovskites. The change in Voc can be attributed to the higher carrier density flux, generated by the increased electric field within the absorber due to the LT structures; akin to the well-known photovoltage increase observed in concentrator PV (CPV) technology under optical concentration factors^53^. This heightened electron density can also saturate some of the defect densities added in the model, leading to an increased quasi-Fermi level separation and thus, Voc. As such, this effect can also be responsible for lower recombination and, consequently, higher FF. Additionally, if the regions of stronger photogeneration within the perovskite are effectively localized near the contacts due to LT-induced constructive interference of scattered light waves, this creates favorable conditions for charge carrier collection, in turn, contributing to an overall improvement in electrical performance.

### Luminescent down-shifting properties in PSCs

#### Optical performance of LDS in PSCs

To determine the effectiveness of the LDS layer in PSCs, we first developed a state-of-the-art LDS material composed of tri-ureasil modified by lanthanides (t-U (5000)/Eu^3+^)^10,51,52,54,55^, whose experimentally measured optical properties are shown in Fig. S4 in SM. The emission spectrum is composed of the typical Eu^3+ 5^D_0_ → ^7^F_0-4_ transitions, which indicate effective energy transfer to the Eu^3+^ ions^10,54,55^. The optical effects of the (t-U (5000)/Eu^3+^) material are also demonstrated in the excitation spectra (Fig. S7 in SM) that reveal three main components, peaking at ~280 nm, ~330 nm, and ~420 nm, mainly ascribed to the hybrid host^56^ and the tta excited states^54,57^, respectively. From the real part of the refractive index shown in Fig. S4a in SM, it was determined that an average refractive index of *n* = 1.5 can be used as a background value to represent the inclusion of the LDS layer over the PSCs during the optical simulations. Namely, since the typical thickness of LDS coatings in practical devices is in the range of several microns (much thicker than the structure of the cells), such a coating was considered in the optical simulations as a background index *n* = 1.5 surrounding the PSCs – instead of a layer on top of the front contact – to account for realistic LDS effects.

**Fig. 4:**
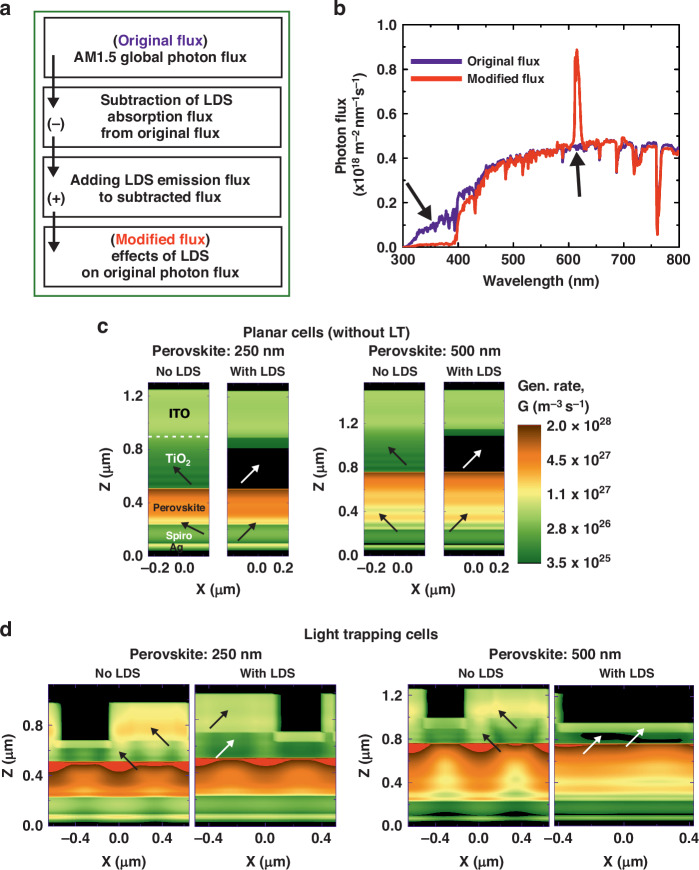
Analysis of LDS improvements on LT-structured and planar PSCs. **a** Diagram of the incorporation method of the LDS effect into the original AM1.5 G photon flux by combining the absorption and emission flux of the LDS material and outputting the LDS-modified flux that will impinge on the solar cells. **b** Original (AM.15 G) and modified photon flux resultant from top-coating the LDS layer made of t-U (500)/Eu^3+^. Log-scale distributions of the optical generation profiles, G, along a cross-sectional plane of the PSCs, for the planar references (**c**) and LT-cells with CB pattern (**d**), with and without the LDS layer of t-U (500)/Eu^3+^ and for distinct perovskite thickness (250 and 500 nm). The arrows highlight the overall LDS effect in PSCs; for instance, in the cases of “No LDS” for planar cells and light trapping cells, the arrows point to high absorption regions in the front contacts (ITO + TiO_2_), whereas in the cases of “With LDS”, the arrow indicates significantly low absorption in the front contacts. Furthermore, for planar cells without LDS, the arrows indicate low absorption in the perovskite, but for LDS, the arrows point strong absorption in the bulk perovskite

As previously explained in the methodologies (see section “Results”), to incorporate the LDS effect of the t-U(5000)/Eu^3+^ material into the simulation, a simple flux conversion scheme is used. This 3-step process was proposed by M. Alexandre et al^18^. and is here summarized in Fig. 4a as well as Fig. S5 in SM. Figure 4b finally compares the converted flux with the original AM1.5G flux.

According to Fig. 4b, the LDS coating shows significant absorption between 300 nm and 400 nm, followed by a clear emission peak at longer wavelengths (600–630 nm). While the profiles also show unwanted parasitic absorption for wavelengths above but close to 400 nm, it is the conversion capabilities of the LDS material that define its final performance. On this note, we calculated 1.14 mA cm^−2^ as the potential maximum contribution to the current if the entire amount of UV radiation (< 400 nm) were down-converted, and 0.73 mA cm^−2^ as the lost contribution to the current due to parasitic absorption by the LDS coating. However, from the emission profile, we only find a photocurrent increase of 0.85 mA cm^−2^ (chiefly at the 600–630 nm peak as shown in Fig. S4c in SM). Therefore, there is still room for improvement in the LDS material, namely by minimizing the absorption beyond 400 nm and maximizing the emission profile in the visible spectrum. Still, in view of PSC application, the amount of UV blockage provided by the LDS coating is, by itself, a promising factor to improve the devices’ stability under illumination, since it can mitigate most of the unwanted UV absorption in the underneath sensitive cell layers, while making partial use of such UV energy that would otherwise be lost.

The optimized cell geometries presented in section “Results” were used to calculate the photocurrent with the original and modified AM1.5G irradiance (see Table S1). For the photonic-enhanced PSCs with LDS layer, the CB geometry was again reoptimized by using the modified AM1.5 G irradiance and the same PSO (Particle Swarm Optimization – see section “Materials and methods”) algorithm for both perovskite thicknesses studied here (250 nm and 500 nm). The results demonstrate that the employment of an LDS layer enables a longer operational lifetime of the PSCs, because of the significantly reduced UV absorption (Fig. 4c, d). Notably, although TiO_2_ experiences almost no parasitic absorption, ITO still shows some at longer wavelengths (Fig. 2a, c). The LDS effect can also be seen in the perovskite layers, especially in the planar cells, as depicted in the photo-generation profiles. In the case with LDS, the light generation in the perovskite layers is slightly higher, as indicated by the arrows, due to the photons converted to the visible wavelengths by t-U (5000)/Eu^3+^. Therefore, our LDS material increases the overall PV performance due to a higher bulk generation through UV photon recycling. Nonetheless, the effectiveness of (t-U(5000)/Eu^3+^) for down-shifting even higher-energy ionizing rays^58^ needs to be evaluated, as it is a further barrier hindering PV implementation in Space.

#### Performance analysis of LT and LDS in PSCs

Aside from its simple geometry making it relevant to industry, another advantage of the current LT scheme over the traditional structuring/roughening approach of absorber layers^34^ is the significant increase in broadband absorption (higher photocurrent), without the typical cost of electrical deterioration due to (surface) defects. This allows the optical benefits to be directly converted into electrical gains, resulting in increased PCE. Furthermore, the addition of an LDS layer improves the UV stability of PSCs and thus has the potential to extend their operational lifetime.

**Fig. 5:**
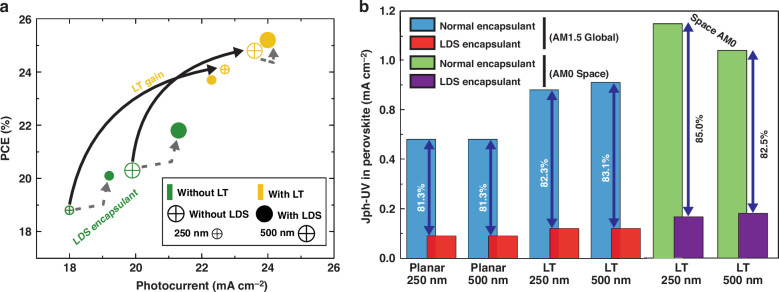
Summary of main results of this study, using original and LDS-modified AM1.5 and AM0 spectra. (**a**) Progression of the PCE and photocurrent values attained with the planar (without LT) and photonic-enhanced (with LT) PSCs, with and without the LDS layer, for 250 nm and 500 nm perovskite thicknesses. **b** Photocurrent contribution generated only by UV light (<400 nm wavelength), for the planar and photonic-enhanced PSCs (under AM1.5 and AM0 illumination), shown here for the cases of a typical encapsulant layer (e.g. ethylene vinyl acetate polymer, EVA, or glass, whose *n* value is around 1.5) and an LDS encapsulant (t-U (5000)/Eu^3+^) layer (modified illumination spectra)

The PCE improvements for PSCs with LT are much higher than those for PSCs with LDS. This is because LT leverages a broadband spectrum, while LDS only works in the narrowband UV portion. Figure 5 compares the predicted PCE of devices with the LT and LDS layers to their corresponding planar cells. For ultrathin 250 nm (superior flexibility) and conventional thick 500 nm perovskites, the relative PCE gain of planar cells with LDS layer was only 6.9% and 3.4%, respectively. In contrast, for 250 nm and 500 nm thick perovskites, the relative PCE gain of PSCs with optimized LT – but without LDS layer – was up to 28% and 22.2%, respectively. Unsurprisingly, the PCE gains attained due to LDS in LT-enhanced PSCs were comparatively low, i.e., no gain for a 250 nm thin perovskite and 1.6% for a 500 nm thick perovskite layer (see the electrical simulation results for LDS-embedded PSCs in section S4 in SM, particularly Fig. S8). Since, in such cases, the optimized CB front structures already maximize the achievable photocurrent gain, there is hardly any scope for optical improvements with LDS. Still, both planar and photonic-enhanced PSCs benefit from the near absolute filtering of UV light. Here, up to 94% of it is blocked by the LDS layer composed of t-U (5000)/Eu^3+^, as demonstrated in Fig. 5b. Therefore, despite its marginal PCE improvement, LDS application remains beneficial for improving the UV stability of PSCs in any case.

The more UV photons the incident spectrum contains, the clearer the potential of LDS integration becomes, for example when considering a space application. To analyze this, we recalculated the spectral responses of PSCs with optimized LT using the AM0 spectrum along with its modification (incorporating the LDS effect), as shown in Fig. S6 in SM. In comparison to the AM1.5 spectrum, the LT-enhanced PSCs in space show increased photocurrent by over 20%. In fact, the AM0 provides an additional 22% incident photon flux when compared to the AM1.5 spectrum. For the cases of LT with LDS, we now consider two different modifications of the AM0 spectrum: first, the same as the AM1.5 modification for LDS, exactly as done before and explained in section “Optical performance of LDS in PSCs”; second, the theoretically-ideal case, where all the UV photon flux, that the LDS layer blocks, is converted into the visible spectrum (see section S3 of SM), resulting in a large peak, as shown in Fig. S6 (ideal method) in SM. As a result, the photocurrent achieved with the ideal method is 10% greater than that with the standard LDS method for both 250 nm and 500 nm perovskites, as shown in the inset table of Fig. S6 in SM. Overall, it is confirmed that the LDS layer blocks an even greater amount of UV radiation under Space illumination conditions, as displayed in Fig. 5b. The LDS layer is therefore able to convert this UV energy almost completely into the usable visible spectrum, resulting in a higher photocurrent.

## Discussion

The study introduces a synergistic photonic approach that leverages the optical effects of an advanced light-trapping (LT) structure and a luminescent down-shifting (LDS) coating. The demonstrated checkerboard (CB) based LT structures integrated with perovskite solar cells (PSC) exhibit a remarkable 25.9% increase in photocurrent, even at oblique angles of incidence. Electrically, the LT-enhanced ultrathin devices, featuring a 250 nm thin perovskite layer, demonstrate a proportional efficiency boost, reaching a projected PCE of up to 24.1% – a gain of approximately 28% over the planar reference cell. The study highlights the robustness of the optimized CB patterns, with photocurrent maxima varying by only 1–3% within *a* ±10% parameter variation of the same CB patterns, ensuring a high degree of resilience against fabrication imperfections.

Moreover, the experimentally developed LDS material (t-U (5000)/Eu^3+^) demonstrates potential benefits mainly in enhancing cell stability without sacrificing PV performance. The LDS material significantly reduces harmful photo-generation in the front TiO_2_ layer and at the perovskite/TiO_2_ interface by ~94%, with a slight increase in photogeneration within the perovskite absorber. Although the LDS effect alone shows more noticeable gains in planar PSCs without LT, the photonic-enhanced PSCs exhibit superior performance under Space illumination conditions. In such a scenario, the LDS material, with enhanced UV blockage and re-emission into the visible range, leads to substantial PV performance improvements. These findings reveal a promising strategy to enhance stability without compromising performance, ultimately maximizing the power-to-weight ratio of PV sources - a critical metric for Space applications.

## Materials and methods

We developed coupled optical and electrical simulations to model the response of PSCs and optimize the CB pattern for LT, as previously shown in Fig. 1. The complex refractive indices of the materials in use were taken from a refractive index database^59^ and experimentally verified literature as shown in Figure S1 in SM, both of which have been also provided in a previous contribution^60^. Here, we take a standard PSC layer structure (Fig. 1a), composed of a common MaPbI_3_ perovskite material, whose spectral response is significant in the 300–800 nm wavelength range.

The optoelectronic simulations were performed in a two-step process. Firstly, the optical (electromagnetic) modeling was carried out via a 3D finite-difference time-domain (FDTD) formalism using Ansys Lumerical FDTD^61^. The selected modeling represents the state-of-the-art method to solve Maxwell’s equations in arbitrary geometries and, particularly, to explore all relevant optical responses for both planar and photonic-enhanced PSCs^34,35,62,63^. This optical solver was combined with a robust population-based stochastic optimization algorithm (Particle Swarm Optimization, PSO) to determine the optimal parameter configuration, i.e., the geometrical CB parameters (as described in Fig. 1b and Table S1 in Supplementary Material, SM) that maximizes the device’s optically-inferred photocurrent. Further details regarding the optical modeling and the PSO method are discussed in previous contributions^50,64^. The second step was the electrical simulation by finite-elements method (FEM) via Ansys Lumerical CHARGE software^60,62^. To perform the electrical simulation, the optical output (photo-generation profile) of both planar and photonic-enhanced PSCs from the 3D FDTD modeling was used as input to compute their PV response, i.e. the current density–voltage (J-V) characteristic curve of the solar cells, as a function of the different perovskite thicknesses studied here. To generate a realistic prediction of the electrical output of the PSCs, a set of electronic transport properties for the bulk and surfaces of the materials was selected according to the state-of-the-art literature^2,49,60,65–68^, which is summarized in Table S2 of SM. A detailed description of the electrical simulation methodology can be found elsewhere ^60^.

After demonstrating the coupled optical and electrical modeling, we carried out the fast Fourier transform (FFT) analysis of the checkerboard patterns investigated in PSCs, in order to comprehend how light is dispersed by different CB patterns. The Fourier-series of a surface structure is determined by its aperture function^40^. In this instance, the aperture function was specified as a binary matrix *A* with non-zero elements (alias aperture) of 1. *A* has as many copies of the unit cell-array in its rows and columns as needed to span an area of ca. 500 µm^2^ considering one pixel covers a 5 nm width in actual space. Once the fast Fourier transform of *e*^*iπA*^ was finished, the fast Fourier-components were determined by shifting the zero-frequency to the array’s center. The results of FFT profiles for various checkerboard patterns are provided in section S2 of SM.

To investigate the effects of an LDS encapsulant, we considered the measured optical properties of a state-of-the-art LDS material (t-U (5000)/Eu^3+^ LDS). Subsequently, to incorporate the LDS effect into the optoelectronic simulations, a conversion procedure was devised to translate the experimentally measured data (absorption and emission profiles), as illustrated in Fig. 4 and Figure S4 in SM. Namely, the absolute absorption – given by 1–10^-A^ (*A* is the absorbance) of the synthesized t-U (5000)/Eu^3+^ LDS material – is multiplied by the solar photon flux (i.e., ASTM G-173 global irradiance spectrum)^8^, yielding the absorption flux of the LDS layer. In the next step, the absorbed flux is subtracted from the original incident spectrum to account for the blocked absorption by the t-U (5000)/Eu^3+^ film. And lastly, the emission flux is added to the modified AM1.5 G spectrum to output the “LDS-converted” spectrum incident on the PSC. Afterwards, the CB pattern of the photonic-enhanced PSCs was again re-optimized in the optical solver using this spectrally-adjusted incident light, recalculating the response of the reference planar cells as well, followed by the electrical simulations of their PV response with the LDS effects.

Lastly, to assess the feasibility of the studied LT plus LDS embedded PSCs for Space application, we calculated the spectral response using the AM0 spectrum for both photonic-enhanced and LDS-embedded PSCs, and compared it to the previously calculated (using AM1.5 G spectrum) results. For comparison, an idealized conversion method was also tested in order to implement the LDS effects in the original spectrum, consisting in the conversion of the entire absorption flux in the UV region to the visible spectrum by the LDS material. This can be considered a theoretically-perfect conversion method when compared to the main method used in this work (the procedure is explained in further detail in section S3 of SM). Ionizing rays, akin to UV radiation, pose another significant concern in Space environments due to their potential to degrade non-resistant materials. Addressing this concern, Lang et al. documented^58,59^ PSCs with enhanced resilience to ionizing radiation, rendering them promising candidates for Space PV. Furthermore, the designed CB-based LT structure presented herein is anticipated to remain uncompromised under the influence of high-energy radiation, ensuring the continued functionality of light diffraction.

## Supplementary information

Supplementary Information for Photon shifting and trapping in perovskite solar cells for improved efficiency and stability
