# Publisher Correction: Photon shifting and trapping in perovskite solar cells for improved efficiency and stability

**DOI:** 10.1038/s41377-024-01637-5

**Published:** 2024-09-20

**Authors:** Sirazul Haque, Miguel Alexandre, António T. Vicente, Kezheng Li, Christian S. Schuster, Sui Yang, Hugo Águas, Rodrigo Martins, Rute A. S. Ferreira, Manuel J. Mendes

**Affiliations:** 1 CENIMAT|i3N, Department of Materials Science, School of Science and Technology, NOVA University of Lisbon and CEMOP/UNINOVA, Campus de Caparica, Caparica, Portugal; 2 Department of Physics and CICECO—Aveiro Institute of Materials, University of Aveiro, Campus Universitário de Santiago, Aveiro, Portugal; 3 Materials Science and Engineering, School for Engineering of Matter Transport and Energy, Arizona State University, Tempe, AZ USA; 4 Department of Physics, University of York, Heslington, York UK

**Keywords:** Solar energy and photovoltaic technology, Optoelectronic devices and components

Correction to: *Light: Science & Applications* 10.1038/s41377-024-01559-2, published online 05 September 2024

During the publication process the affiliation number of the author Sui Yang was missed in the original publication. The affiliation number of the author Sui Yang is ‘3’ and the complete affiliation is shown below:

3 Materials Science and Engineering, School for Engineering of Matter Transport and Energy, Arizona State University, Tempe, AZ, USA.

The original article has been updated. The publisher apologizes to the authors & readers for the inconvenience caused.

