# Supplementary material for: Photon shifting and trapping in perovskite solar cells for improved efficiency and stability
Source: Light Sci Appl. 2024 Sep 5;13:238. doi: 10.1038/s41377-024-01559-2 (PMC11377431; doi:10.1038/s41377-024-01559-2)
Supplement: Supplementary file 1 — Supplementary Information for Photon shifting and trapping in perovskite solar cells for improved efficiency and stability [file 41377_2024_1559_MOESM1_ESM.pdf]

Supplementary Information for

Photon shifting and trapping in perovskite solar cells for improved efficiency and stability

Sirazul Haque[1,2,3,*], Miguel Alexandre[1], António T. Vicente[1], Kezheng Li[4], Christian S. Schuster[4], Sui Yang[3], Hugo Águas[1], Rodrigo Martins[1], Rute A. S. Ferreira[2], Manuel J. Mendes[1,*]

**Corresponding authors: shaque7@asu.edu, mj.mendes@fct.unl.pt*

[1] CENIMAT|i3N, Department of Materials Science, School of Science and Technology, NOVA University of Lisbon and CEMOP/UNINOVA, Campus de Caparica, 2829-516 Caparica, Portugal

[2] Department of Physics and CICECO - Aveiro Institute of Materials, University of Aveiro, Campus Universitário de Santiago, 3810-193 Aveiro, Portugal

[3] Materials Science and Engineering, School for Engineering of Matter Transport and Energy, Arizona State University, Tempe, Arizona 85287, United States

[4] Department of Physics, University of York, Heslington, York, YO10 5DD, UK

S1. Optimal parameter sets and tolerance analysis

The complex refractive index spectra of the materials used in this study was taken from an optical index database [1], with the exception of the $MAPbI_3$ perovskite material which was taken from the work of Eerden et al. [2] The spectra are shown in Figure S1, where the left plot shows the real part of the refractive index (*n*) and the right plot shows the imaginary part (*k*).

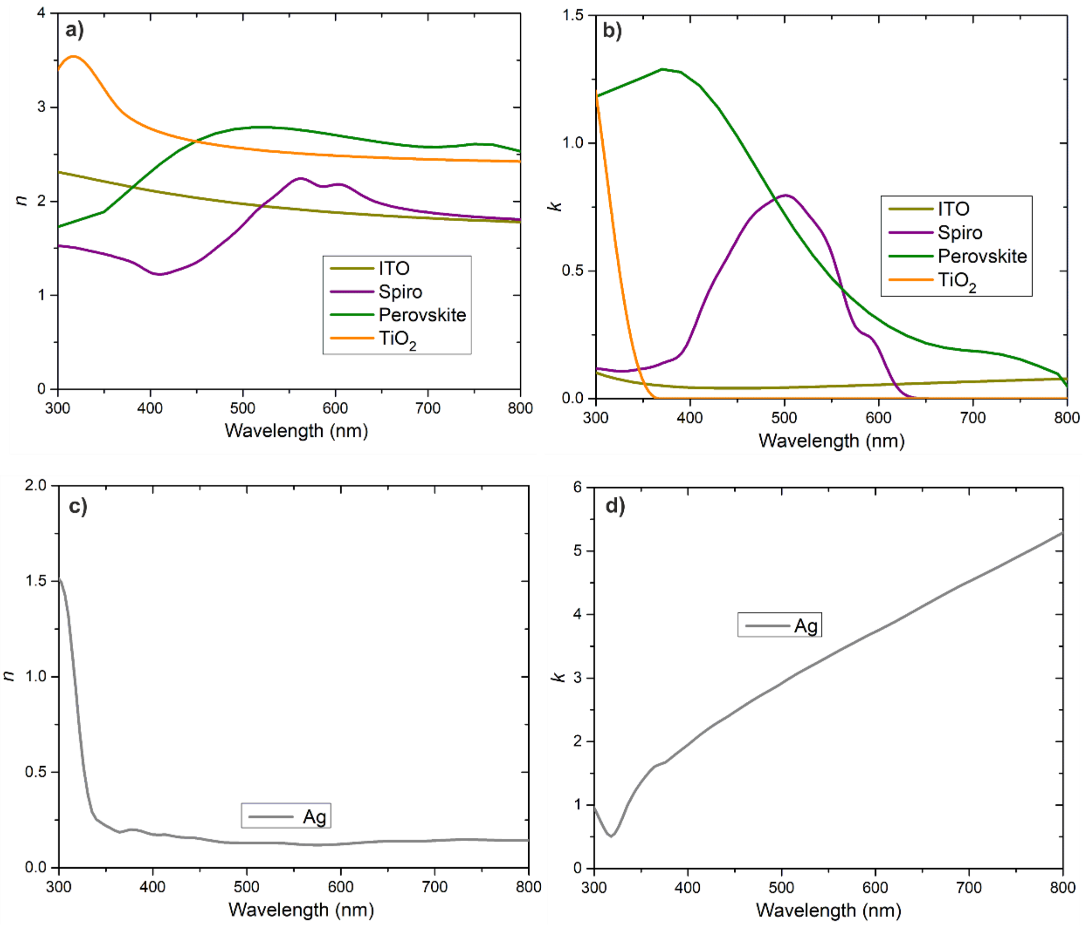

Figure S1: Spectra of the real, *n* (a,c), and imaginary, *k* (b,d), parts of the complex refractive indices of the materials used in this work.

In this work, we consider two different background index values (*n*=1 and *n*=1.5) of practical relevance. The case of *n*=1 corresponds to a substrate-type PSC configuration where the light is incident on the cell-side (transparent conductive oxide, TCO) without an encapsulant layer on the front [8]. Contrastingly, *n*=1.5 emulates the presence of a thick (hundreds of μm) encapsulant film (in this case embedded with luminescent down-shifting,

LDS, fluorophores) applied on the front TCO, since the n value of common encapsulant materials ([e.g., doped Eu^{3+}] is ~1.5.

Table S1: Maximum photocurrent density, J_{PH}, values obtained for the optimized CB arrangement placed on the two distinct PSCs, for 250 nm or 500 nm perovskite layer thickness, with (bottom rows) and without (top rows) an LDS layer, as considered in this work. The geometric optimization parameters (h, w, g, l) of the CB patterns are defined in Figure 1 of the manuscript. To facilitate comparison, the standard thicknesses of the cells' layers (t_{layer}) were kept constant in the optimizations across all cases in the four rows. The thicknesses selected for the HTL (made of Spiro-OMeTAD) and rear contact (made of Ag) align with those commonly employed in state-of-the-art PSCs [3]. The ETL (made of TiO_2) and TCO (made of ITO) are the layers where the checkerboard LT structuring is integrated, with their feasible thicknesses optimized to account for the checkerboard's etching depth (h). The results are compared between the reference planar cell cases and the corresponding CB-patterned (with LT) PSCs, with (bottom rows) and without (top rows) the LDS coating having a refractive index n=1.5. The enhancement values indicated in brackets are the percentual photocurrent gains attained with the CB patterns relative to the planar references.

**Perovskite Solar Cells**		Absorber layer: **250nm perovskite**		Absorber layer: **500nm perovskite**	
		Optimized parameters	J_{PH}, mA cm^{-2} *(enhancement)*	Optimized parameters	J_{PH}, mA cm^{-2} *(enhancement)*
**Uncoated (*n*=1)**	Planar (ref.)	t_{ITO} = 350 nm<br>t_{TiO2} = 400 nm<br>t_{Spiro} = 150 nm<br>t_{Ag} = 100 nm	**18.0**	t_{ITO} = 350 nm<br>t_{TiO2} = 400 nm<br>t_{Spiro} = 150 nm<br>t_{Ag} = 100 nm	**19.9**
	CB pattern (with LT)	h = 283.1 nm<br>w = 84.6 nm<br>g = 577.2 nm<br>l = 648.3 nm	**22.7**<br>*(25.9%)*	h = 251.6 nm<br>w = 153.7 nm<br>g = 532.0 nm<br>l = 653.9 nm	**23.6**<br>*(18.9%)*
**With LDS coating (*n*=1.5)**	Planar (ref.)	t_{ITO} = 350 nm<br>t_{TiO2} = 400 nm<br>t_{Spiro} = 150 nm<br>t_{Ag} = 100 nm	**19.2**	t_{ITO} = 350 nm<br>t_{TiO2} = 400 nm<br>t_{Spiro} = 150 nm<br>t_{Ag} = 100 nm	**21.3**
	CB pattern (with LT)	h = 270.5 nm<br>w = 70.4 nm<br>g = 435.6 nm<br>l = 498.9 nm	**22.3**<br>*(16.1%)*	h = 277.6 nm<br>w = 42.6 nm<br>g = 432.1 nm<br>l = 426.1 nm	**24.0**<br>*(12.7%)*

Table S1 presents the maximum photocurrent density, J_{PH}, values for Perovskite solar cells (PSCs) with two different active layer thicknesses (250 nm and 500 nm) and two optimized architectures, one planar (the control structure) and the other coupled with checkerboard (CB) gratings arranged with symmetry-property that provide light-trapping (LT). Both architectures were also simulated with and without the encapsulant LDS layer. The correlation between the geometrical features of CB patterns and light absorption enhancement is explained in the main manuscript.

The thicknesses taken for the HTL (150 nm, made of Spiro-OMeTAD) and the rear contact (100 nm, made of Ag) are the same for all the analyzed cells and are consistent with those typically employed in state-of-the-art PSCs. For the ETL and TCO, the thicknesses were determined in a prior work specifically dedicated to the optimization of a photonic checkerboard LT structure [4]. As shown in Table S1, the thicknesses for these layers were primarily determined by the checkerboard optimization. In fact, the full ETL thickness (400 nm, made of TiO_2) is relatively high relative to those of state-of-the-art PSCs to account for the checkerboard's etching depth (*h*), which plays a crucial role in the LT performance, while the TCO (350 nm, made of ITO) is assumed to be conformally deposited on top. Given the practical feasibility of the considered thicknesses, and to maintain consistency in our simulations for sake of comparison, we adopted the same thickness values in this work for the planar cases used as reference.

The optimal geometry of CB structures is reasonably tolerant to variations around the optimal parameter sets, as demonstrated by only marginal decreases in the resulting photocurrent density within 20% variation around the optimized values, as shown in Figure S2. This allows for considerably greater freedom in fabricating these CB patterns in real PSC devices.

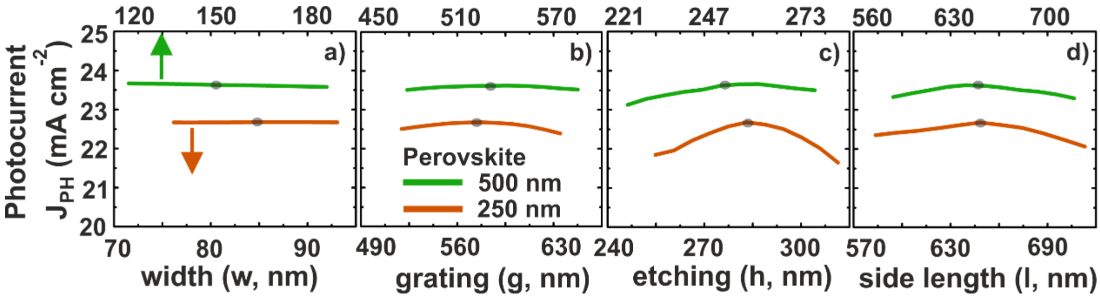

Figure S2: Photocurrent density attained in the LT-enhanced perovskites (without LDS) with a 20% variation (±10% deviation) in each geometrical parameter (*w*, *g*, *h* and *l*) of the CB front structures, relative to the optimal parameter values marked by the dots (indicated in Table S1).

S2. Fast Fourier transform (FFT) analysis

Fourier analysis is commonly used to identify harmonic frequencies in a signal by converting it from the time domain to the frequency domain. In the context of our work, the emphasis is on decomposing 2D geometrical symmetry into the composing 2D

frequencies that represent this symmetry. This process elucidates how a specific light-trapping structure scatters light into different directions, thus helping to identify structures with the greatest potential for optical improvements. The period of the structures is a crucial parameter for coupling surfaces, affecting the optimal coupling band positions in the spectrum. Additionally, the k-space range covered by the coupling surface is also significant, as periodic structures can have highly directional light-trapping performance that may be limiting in some cases. Breaking the symmetry allows for an expansion of the scattering spectrum, enhancing integrated absorption up to a saturation point, typically around 20 μm^{-1} [5]. More information on the mathematical aspects of the FFT procedure can be found elsewhere [6].

In the current analysis, the fast Fourier transform (FFT) of the structures reveals distinct scattering modes, via the off-center peaks in the FFT diagrams (Figure S2a and S2b). Notably, there is a clear preference for light to propagate in two directions, with the structures for the 500 nm perovskite showing a much stronger scattering effect, i.e. stronger FFT off-center peaks. For example, the x factor [4] for 250 nm and 500 nm thick checkerboard patterns is respectively 0.569 and 0.759, with 58.0% and 59.8% of Fourier components localized in $k < 21\ \mu m^{-1}$. Additionally, the number of strong peaks (amplitude higher than 15%) in the 250 nm thick perovskite is 1, while for the 500 nm thick perovskite, it is 21. As indicated in our earlier research [4], the x factor is correlated with the photocurrent density (i.e. short-circuit current), serving as a crucial criterion to assess the light trapping capability of microstructures for thin-film solar cells.

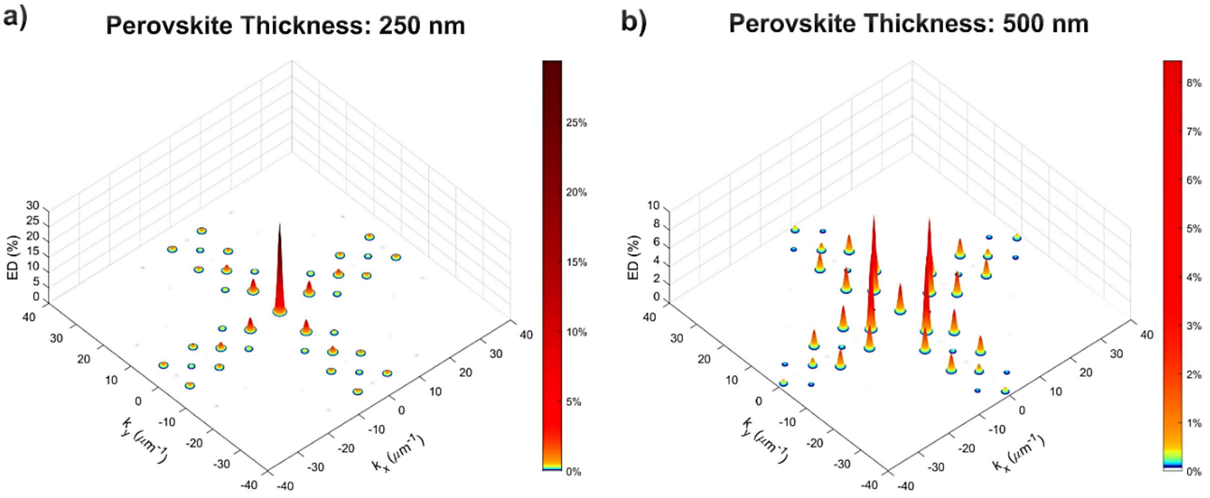

Figure S3: Fast Fourier transform (FFT) analysis showing the scattering modes of the CB patterns in the PSCs (without LDS) for perovskite thickness 250 nm (a) and 500 nm (b). The energy distribution ED(k_x, k_y) is scaled to the total diffracted intensity, which is determined by adding up all of the Fourier components in k space [4]. Each of these structures occupies a real-space area of 500 μm^2. It is processed as a binary data matrix and sampled at a resolution of 5 nm.

S3. The process of converting the experimental properties of t-U (5000)/Eu^{3+} into simulation

In this work, we developed an LDS encapsulant material composed of a tri-ureasil modified by lanthanide ions, namely Eu^{3+} (t-U (5000)/Eu^{3+}). The ureasil is formed by a siliceous-based skeleton covalently grafted to the polymeric chain by urea bridges. The siliceous backbone provides compatibility with current microelectronics and confers enhanced thermal stability (onset of the decomposition temperature at ~230 °C) [7]. The tri-ureasils are processed using environment-friendly green solvents with tunable viscosity, which makes them ideal low-cost inks to be easily printed on virtually any substrate (paper, plastic, and textile) and compatible with printable electronic circuits [7].

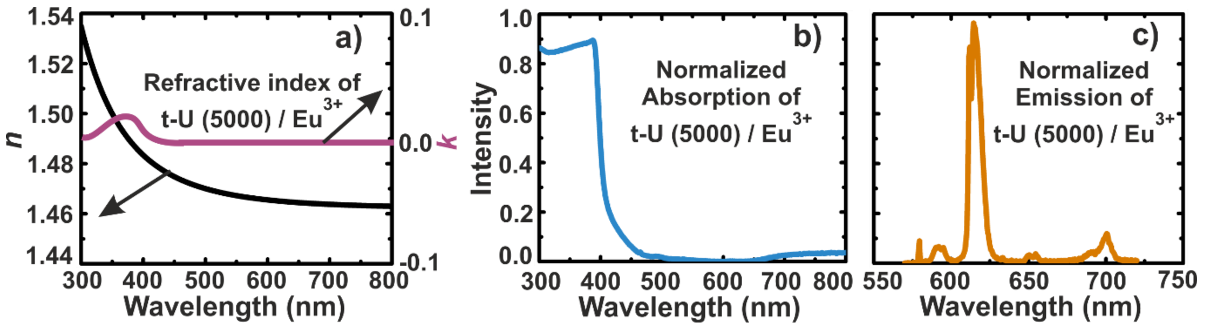

Figure S4: Optical properties of the experimentally developed LDS material, t-U (500)/Eu^{3+}: complex refractive index (a), normalized absorption (b) and normalized emission spectra (c). Adapted from ref [8].

Figures S4b,c show the experimental absorption and emission profiles for t-U (5000)/Eu^{3+}, respectively, being the emission spectrum composed of the typical Eu^{3+} $^5D_0 \rightarrow ^7F_{0-4}$ transitions, as studied elsewhere [8]. The spectral characteristics of the t-U (5000)/Eu^{3+} LDS coatings allow for anticipating UV filtering in combination with downshifting to spectral regions where the PSCs exhibit higher spectral response.

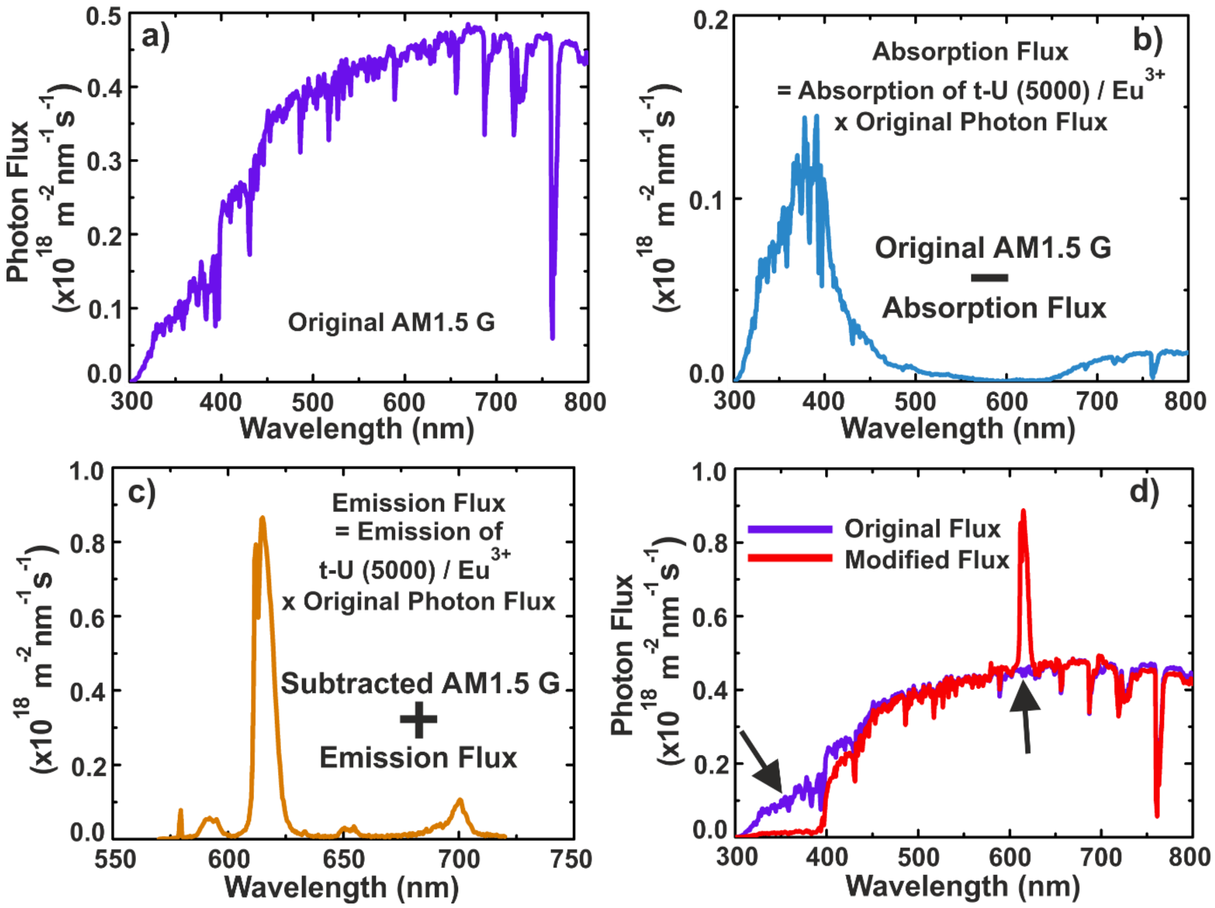

Figure S5: Representation of the method used to emulate the process of the down-shifting feature of t-U (500)/Eu^{3+} in PSCs. a) original AM1.5 G photon flux, b) subtracted AM1.5 G photon flux obtained by subtracting the Absorption flux of LDS t-U (5000)/Eu^{3+} from the original one, c) emission flux of LDS t-U (5000)/Eu^{3+} added to the subtracted AM1.5 G photon flux, and d) comparison of the original AM1.5 G photon flux with the resulting LDS-modified AM1.5 G photon flux.

The process flow of translating the experimental properties of the LDS t-U (5000)/Eu^{3+} into the simulations is shown in Figure S5. Firstly, the absorption flux of t-U (5000)/Eu^{3+} is subtracted from the original AM1.5G flux, followed by the addition of the emission flux of t-U (5000)/Eu^{3+}. This way the LDS effect is accounted for in the resulting modified spectrum incident on the PSCs.

To analyze the potential impact that the LDS effect could have in the modeling system, in addition to the above method we tested a conceptual ideal case in which the total UV photon flux blocked by the LDS encapsulant is transformed to the visible spectrum as shown in Figure S6.

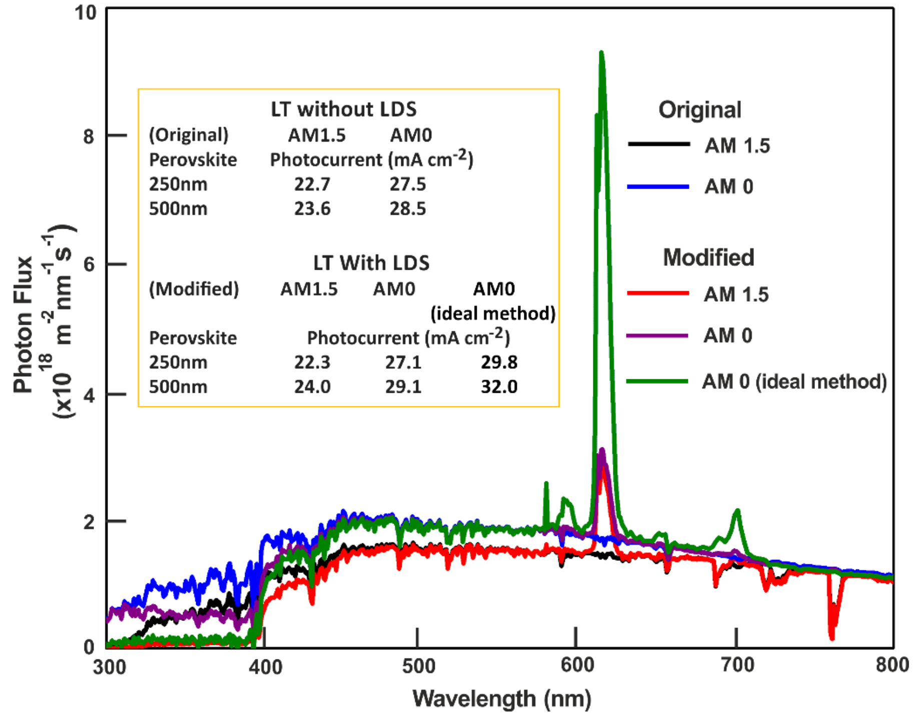

Figure S6: Original and LDS-modified (considering down-shifting effect of t-U (500)/Eu^{3+}) AM1.5 and AM0 irradiance spectra. The inset table shows the photocurrent density values attained with both spectra for the PSCs with LT and LT plus LDS, with distinct perovskite layer thicknesses.

The absence of intrinsic emission from ligands and hybrid excited states indicates effective energy transfer to the Eu^{3+} ions, as shown in the excitation spectra, which reveal three main components, peaking at 280 nm, 330 nm, and 420 nm, as shown in Figure S7, which are primarily attributed to the hybrid host [9] and to tta excited states [10,11], respectively. The 330 nm and 420 nm components are similar to those previously found for isolated $Eu(tta)_3.2H_2O$ [12] and for organic-inorganic hybrids incorporating Eu^{3+} complexes, being ascribed to the π-π* electronic transition of the organic ligands [13]. Aside from changes in relative intensity, the absorption spectrum shows the same components as the excitation spectra [2,8].

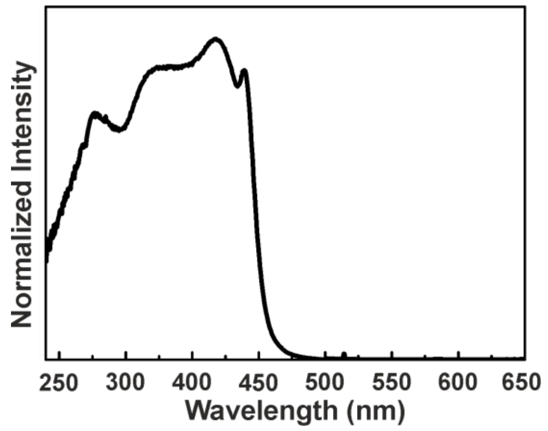

Figure S7: Excitation spectrum monitored at 612 nm for the t-U (5000)/Eu^{3+}.

S4. Electrical modelling

Table S2 shows the physical properties that were employed for the electrical simulations in this work, presented in section 3.1.2 of the manuscript. These parameters are consistent with those found in experimental observations, as explained in further detail in a previous contribution [3].

Table S2: Parameters used for the electrical simulations.

Material	Parameters	Value	Unit
**Perovskite Absorber**	Conduction band minimum	-4.0	eV
	Valence band maximum	-5.6	eV
	Bandgap	1.6	eV
	Effective density of states of conduction band	$5\text{x}10^{18}$	cm^{-3}
	Effective density of states of valence band	$5\text{x}10^{18}$	cm^{-3}
	Dielectric permittivity (relative)	25	
	Electron mobility	10	$cm^2\ (Vs)^{-1}$
	Hole mobility	10	$cm^2\ (Vs)^{-1}$
	Radiative recombination coefficient	10^{-11}	$cm^3\ s^{-1}$
	SRH lifetime bulk	$2.5\text{x}10^{-7}$	s
**HTL/ETL Interfaces with Absorber**	SRH surface recombination velocity, S_0	140	$cm\ s^{-1}$

Figure S8 shows the current-voltage curves attained for planar and photonic-enhanced PSCs, both with the previously mentioned LDS layer composed of t-U (5000)/Eu^{3+}. These results are discussed in Section 3.2.2 of the main manuscript.

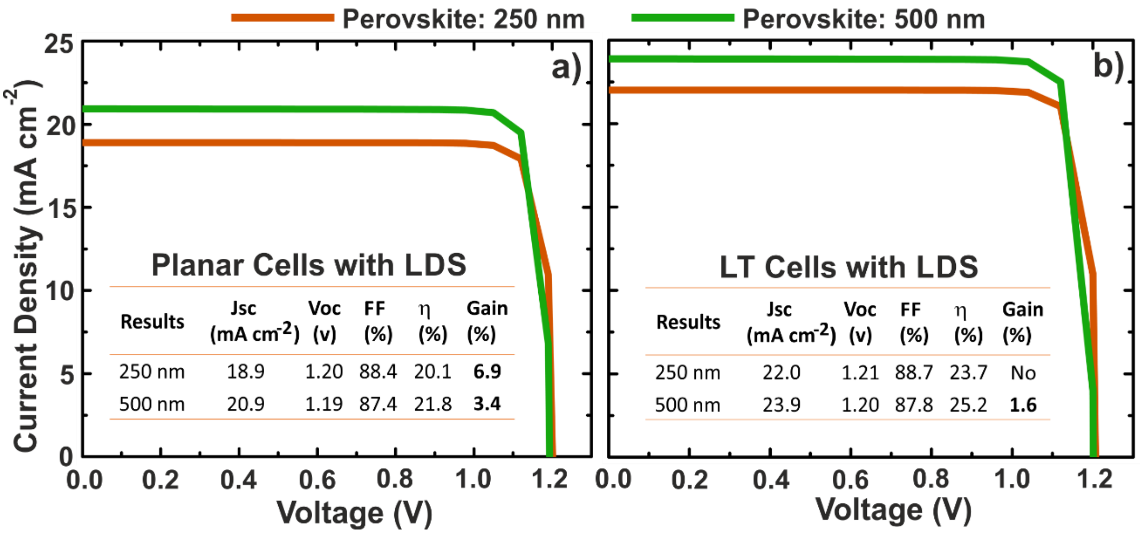

Figure S8: Electrical simulation results for planar (a) and photonic-enhanced (b) PSCs with the LDS layer, for 250 nm and 500 nm perovskite thickness. The inset tables show the PV characteristics, where the values of the PCE gains solely due to the LDS effect in each case are indicated.

References

[1] Refractive index database, (n.d.). https://refractiveindex.info/ (accessed June 1, 2022).

[2] M. van Eerden, M. Jaysankar, A. Hadipour, T. Merckx, J.J. Schermer, T. Aernouts, J. Poortmans, U.W. Paetzold, Optical Analysis of Planar Multicrystalline Perovskite Solar Cells, Adv. Opt. Mater. 5 (2017) 1700151. https://doi.org/10.1002/adom.201700151.

[3] S. Haque, M. Alexandre, C. Baretzky, D. Rossi, F. De Rossi, A.T. Vicente, F. Brunetti, H. Águas, R.A.S. Ferreira, E. Fortunato, M.A. der Maur, U. Würfel, R. Martins, M.J. Mendes, Photonic-Structured Perovskite Solar Cells: Detailed Optoelectronic Analysis, ACS Photonics. (2022). https://doi.org/10.1021/ACSPHOTONICS.2C00446.

[4] K. Li, S. Haque, A. Martins, E. Fortunato, R. Martins, M.J. Mendes, C.S. Schuster, Light trapping in solar cells: simple design rules to maximize absorption, Opt. Vol. 7, Issue 10, Pp. 1377-1384. 7 (2020) 1377–1384. https://doi.org/10.1364/OPTICA.394885.

[5] J. Li, K. Li, C. Schuster, R. Su, X. Wang, B.H. V. Borges, T.F. Krauss, E.R. Martins, Spatial resolution effect of light coupling structures, Sci. Reports 2015 51. 5 (2015) 1–8. https://doi.org/10.1038/srep18500.

[6] E.R. Martins, J. Li, Y. Liu, V. Depauw, Z. Chen, J. Zhou, T.F. Krauss, Deterministic quasi-random nanostructures for photon control, Nat. Commun. 2013 41. 4 (2013) 1–7. https://doi.org/10.1038/ncomms3665.

[7] J.F.C.B. Ramalho, S.F.H. Correia, L. Fu, L.M.S. Dias, P. Adão, P. Mateus, R.A.S. Ferreira, P.S. André, Super modules-based active QR codes for smart trackability and IoT: a responsive-banknotes case study, Npj Flex. Electron. 2020 41. 4 (2020) 1–9. https://doi.org/10.1038/s41528-020-0073-1.

[8] S.F.H. Correia, A.R.N. Bastos, L. Fu, L.D. Carlos, P.S. André, R.A.S. Ferreira, S.F.H. Correia, A.R.N. Bastos, L. Fu, L.D. Carlos, P.S. André, R.A.S. Ferreira, Lanthanide-based downshifting layers tested in a solar car race, Opto-Electronic Adv. 2 (2019)

190006–1. https://doi.org/10.29026/OEA.2019.190006.

[9] V.T. Freitas, P.P. Lima, R.A.S. Ferreira, E. Pecoraro, M. Fernandes, V. De Zea Bermudez, L.D. Carlos, Luminescent urea cross-linked tripodal siloxane-based hybrids, J. Sol-Gel Sci. Technol. 65 (2013) 83–92. https://doi.org/10.1007/S10971-012-2770-2/FIGURES/7.

[10] C. Molina, K. Dahmouche, Y. Messaddeq, S.J.L. Ribeiro, M.A.P. Silva, V. De Zea Bermudez, L.D. Carlos, Enhanced emission from Eu(III) β-diketone complex combined with ether-type oxygen atoms of di-ureasil organic–inorganic hybrids, J. Lumin. 104 (2003) 93–101. https://doi.org/10.1016/S0022-2313(02)00684-1.

[11] M.M. Nolasco, P.M. Vaz, V.T. Freitas, P.P. Lima, P.S. André, R.A.S. Ferreira, P.D. Vaz, P. Ribeiro-Claro, L.D. Carlos, Engineering highly efficient Eu(III)-based tri-ureasil hybrids toward luminescent solar concentrators, J. Mater. Chem. A. 1 (2013) 7339–7350. https://doi.org/10.1039/C3TA11463E.

[12] J. Kai, M.C.F.C. Felinto, L.A.O. Nunes, O.L. Malta, H.F. Brito, Intermolecular energy transfer and photostability of luminescence-tuneable multicolour PMMA films doped with lanthanide–β-diketonate complexes, J. Mater. Chem. 21 (2011) 3796–3802. https://doi.org/10.1039/C0JM03474F.

[13] M. Fernandes, V. De Zea Bermudez, R.A. Sá Ferreira, L.D. Carlos, A. Charas, J. Morgado, M.M. Silva, M.J. Smith, Highly photostable luminescent poly(ε-caprolactone)siloxane biohybrids doped with europium complexes, Chem. Mater. 19 (2007) 3892–3901. https://doi.org/10.1021/CM062832N/SUPPL_FILE/CM062832NSI20070419_060640.PDF.
